# Left Atrium and Left Atrial Appendage Screening Prior to Atrial Fibrillation Ablation: A Comprehensive Review of the Literature

**DOI:** 10.19102/icrm.2018.090502

**Published:** 2018-05-15

**Authors:** Nicholas J. Serafini, Kousik Krishnan

**Affiliations:** ^1^Division of Electrophysiology, Rush University Medical Center, Chicago, IL, USA

**Keywords:** Atrial fibrillation, catheter ablation, left atrial thrombus, transesophageal echocardiogram

## Abstract

In the past decade, certain patient groups have been observed to have a presumptive lower incidence of left atrial (LA)/LA appendage (LAA) thrombus, particularly those who have no or minimal comorbidities. This has led to the question of whether a preprocedural evaluation of the LA/LAA is necessary in every patient, or if it can be avoided in certain patient populations. The answer to this is further complicated by the possibility of newer anticoagulation approaches affecting the incidence of intracardiac thrombus, including uninterrupted warfarin therapy and uninterrupted novel oral anticoagulant (NOAC) therapy, both of which are becoming more common. In this study, we conducted a comprehensive review of the literature addressing these questions in an attempt to summarize current approaches to evaluating the LA/LAA prior to ablation in order to elucidate the strategies that are currently being employed, to determine which strategies are becoming more favorable for use, and to identify what topics can or should be targeted for future study. In addition, this paper seeks to address the following specific questions: is ruling out LA/LAA thrombus necessary in all patients prior to atrial fibrillation (AF) ablation? Are there any identifiable patient populations at reliably lower risk who could potentially avoid LA/LAA thrombus screening prior to AF ablation? What are the current strategies being employed by electrophysiologists in the published literature? What is the opinion of the current literature on warfarin and NOAC drugs as they pertain to the incidence of LA/LAA thrombus prior to AF ablation, and how does each fit into the current treatment strategies for the prevention of procedural thromboembolism? Finally, what is the future of preprocedural intracardiac thrombus evaluation prior to AF ablation, and what steps can be taken to ensure that the risk to the patient is minimized while improving laboratory efficiency and avoiding unnecessary costs?

## Introduction

**Figure 1:**
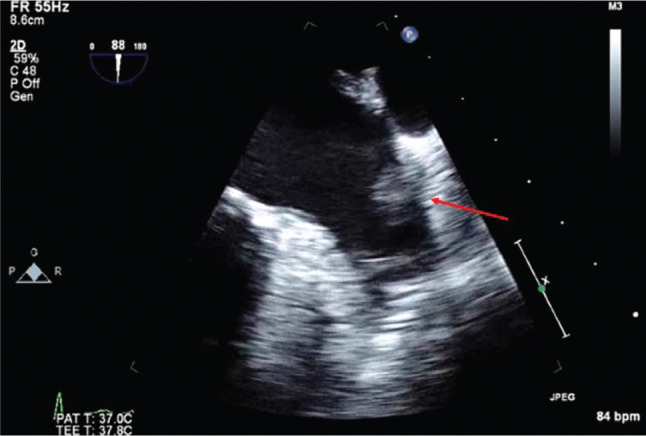
A representative example of a patient with paroxysmal AF presenting in sinus rhythm with a high CHA_2_DS_2_-VASc score = 5 (CHADS_2_ score = 3) on uninterrupted Coumadin^®^ (warfarin; Bristol-Myers Squibb, New York, NY, USA) with a therapeutic international normalized ratio found to have a preprocedural LAA thrombus using TEE. The arrow depicts the LAA thrombus. The thrombus appeared resolved on a subsequent TEE scan after six weeks of escalated intensity of warfarin therapy, and the patient subsequently underwent a successful AF ablation procedure without complication.

**Figure 2:**
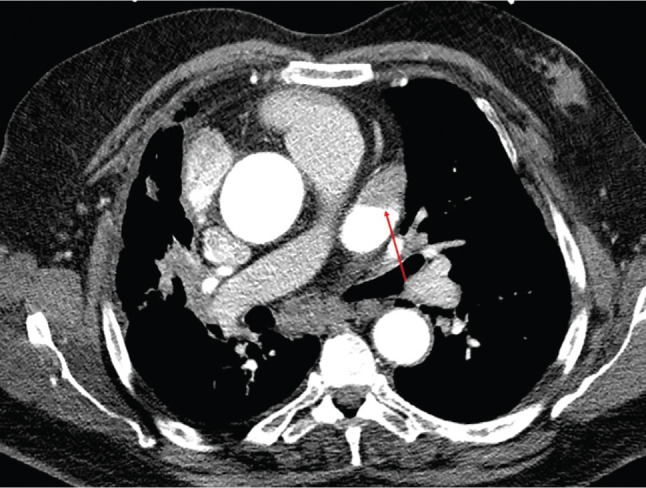
A patient with persistent AF and a CHA_2_DS_2_-VASc score = 4 found to have a filling defect on cardiac CT suggestive of LAA thrombus. The red arrow points out the LAA thrombus.

Thromboembolic prophylaxis management in the peri-procedural period has an integral role in minimizing embolic complications during atrial fibrillation (AF) ablation. As more knowledge has been gained about the individual complications of AF ablation, strategies (eg, the avoidance of ablation inside the pulmonary veins, thereby minimizing the risk for pulmonary stenosis) have been developed to minimize and, in some cases, nearly eliminate what were once common occurrences. Efforts to understand the various mechanisms of procedure-related embolic complications—including (1) prior intracardiac thrombus; (2) air introduction during transseptal access and catheter/sheath exchange; (3) thrombus and char formation on the ablation catheter’s tip; and (4) thrombus formation due to endothelial damage at the site of ablation contact^1,2^—have led to techniques that have markedly reduced thromboembolic complications, such as targeting an activated clotting time level greater than 300 seconds^2^; using irrigated-tip radiofrequency catheters to avoid char; and taking meticulous care of the catheters to avoid the introduction of air during left atrium (LA) access, particularly during catheter exchanges. In patients presenting for AF ablation, preexisting LA appendage (LAA) thrombus occurs in up to 11.7%^3^ and is an absolute contraindication for instrumentation application in the LA. Transesophageal echocardiography (TEE) has evolved as the gold standard of LA/LAA thrombus identification, as seen in **Figure 1**; however, other modalities are routinely employed, such as contrast-enhanced multidetector computed tomography (ce-MDCT) with high sensitivity, as seen in **Figure 2**. In the past decade, certain patient groups have been observed to have a presumptive lower incidence of LA/LAA thrombus, particularly those who have no or minimal comorbidities. This has led to the question of whether a preprocedural evaluation of the LA/LAA is necessary in every patient, or if it can be avoided in certain patient populations. The answer to this is further complicated by the possibility of newer anticoagulation approaches affecting the incidence of intracardiac thrombus, including uninterrupted warfarin therapy and uninterrupted novel oral anticoagulant (NOAC) therapy, both of which are becoming more common.^4^ While very safe, methods to exclude LAA thrombus introduce small but finite risk, expense, and operational inefficiencies.

In this study, we conducted a comprehensive review of the literature addressing these questions in an attempt to summarize current approaches to evaluating the LA/LAA prior to ablation, to elucidate the strategies that are being employed at this time, to determine which strategies are becoming more favorable for use, and to identify what topics can or should be targeted for future study. In addition, we aimed to address the following specific questions: is ruling out LA/LAA thrombus necessary in all patients prior to AF ablation? Are there any identifiable patient populations at reliably lower risk who could potentially be exempt from undergoing LA/LAA thrombus screening prior to AF ablation? What are the current strategies employed amongst electrophysiologists in the published literature? What is the opinion of the current literature on warfarin and NOAC drugs as they pertain to the incidence of LA/LAA thrombus prior to AF ablation, and how does each fit into current treatment strategies for the prevention of procedural thromboembolism? Finally, what is the future of preprocedural intracardiac thrombus evaluation prior to AF ablation, and what steps can be taken to ensure that the risk to the patient is minimized while improving laboratory efficiency and avoiding unnecessary costs?

## Materials and methods

A review of the literature included PubMed searches for the following terms “trans(o)esophageal echocardiogram/echocardiography,” “computed tomography/CT,” “left atrial appendage thrombus,” “left atrial thrombus,” and “atrial fibrillation ablation” through May 2017. An individual screening of each result was conducted and papers were included based on their relevance to the questions cited in the Introduction section. If a paper was deemed relevant by the authors, a review of the entire bibliography of each paper was conducted and additional papers were included in the current study based on their determined relevance. The included studies can be found in **Table 1**.

## Findings of the literature search

### Identification of patient populations and anticoagulation approaches to nonvalvular atrial fibrillation ablation

As the volume of AF ablation has increased, a number of small cohort studies and several case series have suggested the presenting rhythm, risk factors for thrombus formation, and the duration of anticoagulation are all factors that are intimately involved in the prevalence of LA/LAA thrombus prior to AF ablation. As such, several different groups of patients can be identified in an attempt to stratify their individual risk of presenting with an LA/LAA thrombus, including (1) patients who present in sinus rhythm with minimal risk factors who are not anticoagulated prior to AF ablation; (2) those who present in sinus rhythm having been on more than three weeks of anticoagulation therapy; (3) those who present in sinus rhythm having been on less than three weeks of anticoagulation therapy; (4) those who present in AF having been on more than three weeks of anticoagulation therapy; and (5) those who present in AF with less than three weeks of anticoagulation therapy. Each of these patient groups can be further stratified based on their associated risk profile for LA/LAA thrombus formation, which includes the utilization of the guidelines-recommended CHA_2_DS_2_-VASc scoring system^4^ and more novel risk factors such as diastolic function, renal failure, LA size, the presence of structural heart disease, B-type natriuretic peptide level, and type of AF.

Adding additional complexity to stratifying each of these groups, the anticoagulation approaches have changed dramatically in the past decade, from initially stopping oral anticoagulation (OAC) prior to ablation with or without low-molecular-weight heparin bridging to continuing warfarin therapy uninterrupted and, more recently, to continuing NOAC therapy uninterrupted (or withholding only one or two NOAC doses). Notably, however, the impact of these latter approaches on the incidence of LA/LAA thrombus prior to AF ablation remains unclear. The writing committee for the latest joint Heart Rhythm Society/European Heart Rhythm Association/Latin American Society of Electrophysiology and Cardiac Stimulation/Asia Pacific Heart Rhythm Society/European Cardiac Arrhythmia Society (HRS/EHRA/SOLAECE/APHRS/ECAS) expert consensus statement on AF ablation reported that 51% of the committee performs TEE in all patients regardless of their presenting rhythm or anticoagulation status, highlighting that the approaches to excluding LA/LAA thrombus evaluation remain highly variable.

### Identifying patient-specific risk factors in patients presenting for atrial fibrillation ablation

Identifying patient-specific risk factors for LA/LAA thrombus with high positive predictive power has been a challenge. Several scoring systems have been proposed, with the CHA_2_DS_2_-VASc system emerging as the guidelines-recommended approach^4^ to risk stratification for predicting thromboembolic risk in the general nonvalvular AF population. The majority of studies analyzing patients who present for AF ablation find higher LA/LAA thrombus rates in patients with either higher CHA_2_DS_2_-VASc/CHADS_2_ scores or those who are presenting in AF, with rates incrementally increasing as the CHA_2_DS_2_-VASc (or CHADS_2_ in earlier studies) scores increase.^5,6^ Furthermore, patients who present for AF ablation in AF were found to have a higher likelihood for the presence of LA/LAA thrombus.^7–11^ Conflicting data exist in patients presenting in sinus rhythm with low CHA_2_DS_2_-VASc (or CHADS_2_) scores. In a recent Italian multicenter retrospective analysis of 1,539 patients, 12 patients were found to have LA/LAA thrombus. Of those patients who had LA/LAA thrombus and presented in sinus rhythm, all had CHA_2_DS_2_-VASc scores ≥ 2. Those with LA/LAA thrombus who had a CHA_2_DS_2_-VASc score of 0 or 1 all presented in AF.^2^ In 2011, in a prospective study involving 408 patients referred for AF ablation, all of whom were anticoagulated beforehand with warfarin and bridged with low-molecular-weight heparin prior to the procedure, there were six cases of LA/LAA thrombus found, with five of them occurring in patients with CHADS_2_ scores < 2. All of the patients with LAA thrombus had persistent AF with LA dilation, both of which were independent predictors along with female gender, of LA/LAA thrombus in this study.^8^ In a retrospective cohort study of 198 patients, seven of the 198 subjects had LA/LAA thrombus. Three of these seven patients had normal left ventricular function with paroxysmal AF, while two of the seven had paroxysmal AF and presented in sinus rhythm, both with a CHADS_2_ score = 2.^12^ Similarly, in two additional cohort studies, a very low incidence of LA/LAA thrombus was found in those patients with minimal risk factors.^10,11^ In a Polish study of 151 consecutive patients, a marked 10% LA/LAA thrombus or dense smoke (ie, spontaneous echo contrast) rate was found, with persistent AF and an estimated glomerular filtration rate of < 50 ml/min/1.73 m^2^ being independent predictors on multivariate logistic regression analysis. The receiver operating characteristic curves favored adding renal dysfunction and type of AF to the CHA_2_DS_2_-VASc scoring system, although without statistical significance.^6^

Attempting to further advance risk stratification in AF ablation candidates, the identification of alternative risk factors for LA/LAA thrombus in all subjects with nonvalvular AF may be applicable in those patients presenting for AF ablation. Recently, diastolic dysfunction with high filling pressures has been elucidated as providing incremental predictive value to the CHA_2_DS_2_-VASc scoring system.^13^ In addition to the aforementioned small study that found renal dysfunction to be an independent predictor of LA/LAA thrombus,^6^ a large meta-analysis (conducted irrespective of AF ablation intention) of 538,479 patients and 42,719 thromboembolic events identified renal disease as a significant additional predictor of LA/LAA thrombus.^14^ Whereas none of these risk factors carry a high positive predictive power for the presence of thrombus in the setting of AF ablation, their absence may play a role in identifying those who are at very low risk of such.

### Role of the preablation anticoagulation strategy

Further confounding accurate risk stratification, pre-ablation anticoagulation strategies play a role in the prevalence of LA/LAA thrombus in those individuals presenting for AF ablation. The concept of performing AF ablation on uninterrupted anticoagulation was reported in 2007^15^ in a study comparing uninterrupted warfarin to two different strategies of anticoagulation management prior to AF ablation (bridging with high-dose low-molecular-weight heparin and low-dose low-molecular-weight heparin, respectively), which found no increase in major complications and a decrease in bleeding rates and significantly less spontaneous echo contrast in the uninterrupted warfarin group. In a separate 2010 study, however, the rates of LAA thrombus despite adequate OAC remained elevated (affecting seven of 192 patients, or 3.6%) in patients presenting for AF ablation on four weeks of uninterrupted warfarin therapy.^12^ Current clinical approaches and accepted guidelines for the cardioversion of AF lasting more than 48 hours in duration to prevent thromboembolic complications allow for either a TEE scan or three weeks of uninterrupted, therapeutic anticoagulation, suggesting the possibility that thromboembolism is unlikely to occur in this group. One must be vigilant in understanding the fundamental difference in instrumentation of the LA and possible LAA during AF ablation versus electrical cardioversion, however. Although applicable to cardioversion therapy, this strategy does not assess the prevalence of LA/LAA thrombus in the cohort, a component critically important to the electrophysiologist prior to instrumentation of the LA.

In line with uninterrupted warfarin, uninterrupted dabigatran and subsequently rivaroxaban have been compared with uninterrupted warfarin, each demonstrating comparable risk of embolic events with possibly decreased bleeding complications.^16–26^ In a 2013 study involving 999 consecutive patients undergoing AF ablation, researchers compared those on uninterrupted warfarin to those on dabigatran 150 mg twice a day (with one to two doses withheld preablation), who were propensity matched to controls, and found no difference in thromboembolic or bleeding complications.^16^ Similarly, in meta-analyses comparing the use of dabigatran to uninterrupted warfarin, similar rates of thromboembolic and bleeding were observed in the majority of cases.^18–20^ In 2014, a large, retrospective single-center analysis involving 1,745 patients with different preablation anticoagulation regimens showed overall very low major bleeding rates or thromboembolic rates when comparing the use of warfarin plus low-molecular-weight heparin bridging with aspirin, dabigatran with no bridging, and rivaroxaban with no bridging pre-AF ablation. All patients underwent preprocedural TEE. An observed 1.63% LA/LAA thrombus rate was seen, with a statistically higher rate present in the rivaroxaban group, although the authors pointed out that the majority of these individuals stopped rivaroxaban earlier than recommended.^20^ On the contrary, in the recent Uninterrupted Dabigatran Etexilate in Comparison to Uninterrupted Warfarin in Pulmonary Vein Ablation (RE-CIRCUIT) trial, all participants received documented therapeutic OAC or dabigatran for four weeks to eight weeks prior to AF ablation, and doses were also given the morning of the procedure. Of 635 randomized patients, one subject’s procedure was cancelled due to LA thrombus, although the mean CHA_2_DS_2_-VASc scores were low.^26^

As more data continue to emerge that point out the decreased bleeding complications associated with uninterrupted warfarin, more electrophysiologists are performing AF ablation with such a strategy. In the latest joint HRS/EHRA/SOLAECE/APHRS/ECAS expert consensus statement on AF ablation, in a survey of committee writing members, the vast majority (87%) reported performing AF ablation on uninterrupted warfarin in those who present on warfarin therapy. Performing procedures on uninterrupted NOAC therapy had substantially lower response rates. Still, there is a growing number of electrophysiologists who are employing NOAC drugs as a preablation anticoagulation strategy.

Additionally, a prospective analysis of 197 patients compared three weeks of therapeutic OAC with warfarin plus bridging with low-molecular-weight heparin to no OAC therapy pre-AF ablation and found a significantly reduced incidence of LA/LAA thrombus (11.7% versus 6.3%)^3^—an additional testament to the high rate of LA/LAA thrombus that can present despite the institution of a rigorous three-week OAC program beforehand.

Taken together, these data support the paradigm of an increasing number of ablation procedures taking place on NOAC drugs, either uninterrupted or with one to two doses withheld, but do not show clear evidence that these drugs obviate the need for LA/LAA thrombus screening beyond the aforementioned presenting rhythm and risk factor stratification.

## Cost-effectiveness of transesophageal echocardiography prior to atrial fibrillation ablation

In a simulated decision analysis model using Markov methodology to generate incremental costs per quality-adjusted life year (QALY), for routine use of TEE without risk stratification, the cost per QALY was $226,608. In high-risk simulated patients, the incremental cost per QALY was $2,232.^27^ While this cannot be taken as justification to forgo LA/LAA thrombus screening prior to AF ablation in lower-risk patients, it demonstrates that the current approaches being used in screening may need modification.

### Alternative strategies: computed tomography, intracardiac echocardiography, and magnetic resonance imaging

It should be noted that, although the added risk to the patient is low when preprocedural TEE is included in the AF ablation procedure (0.3%),^28^ there is added cost (as previously noted) and laboratory efficiency may be hindered. In the past 15 years, several additional tools have become available to the electrophysiologist for use in looking for the presence of cardiac thrombus prior to AF ablation, including intracardiac echocardiography (ICE), delayed ce-MDCT, and cardiac magnetic resonance imaging.

Cardiac ce-MDCT is a promising modality as an alternative to TEE in screening the LAA. Although initial studies showed promise with its use, a significant number of “pseudo-filling defects” have since been detected in other studies, which are attributed to first-pass scanning and/or variable scan quality with inadequate scan resolution. As computed tomography (CT) scanning technology has advanced, the ability to accurately identify either forthright LA/LAA thrombus or a high suspicion for such has markedly improved.^29,30^ In a recent prospective study, a 60-second delayed-phase scan of the LA/LAA was obtained in addition to the standard first-phase scan, which the patients were already receiving as part of preprocedural anatomic planning.^31^ With adding only a small amount of additional radiation (0.4 mSv on average), they were able to resolve 17 of 20 filling defects as “pseudo-filling defects” when compared with first-phase scanning. Three of 120 patients had true LAA thrombus, as confirmed using TEE. An impressive ability to decrease the number of TEE scans ordered before AF ablation by integrating a delayed phase in patients already undergoing a contrast-enhanced CT scan for pulmonary vein anatomy was also recently reported.^29^ An abnormal delayed, contrast-enhanced CT scan prompted a confirmatory TEE, and high-risk patients with negative delayed ce-MDCT were recommended to undergo TEE. No high-risk patients with a negative delayed ce-MDCT finding demonstrated thrombus. As the majority of patients receive a pre-procedural ce-MDCT for pulmonary vein anatomy, the incorporation of a delayed phase for identifying filling defects may become a useful tool in ruling out thrombus, as long as timing and anticoagulation management bridge the time from the scan to the procedure.

In a similar fashion to delayed ce-MDCT, ICE has emerged as a viable modality to identify thrombus within the left atrium and pulmonary veins. When analyzing retrospective data looking at those who underwent preprocedural TEE and intraprocedural ICE imaging, ICE was able to identify thrombus in seven of 122 patients who were either negative or inconclusive on TEE scan.^32^ In a separate study, seven of nine patients who were identified as “equivocal” or as demonstrating “high suspicion” for LAA thrombus on TEE were resolved by ICE imaging, with subsequent successful completion of AF ablation in those patients.^33^ ICE imaging is, however, subject to a high degree of variability amongst those manipulating the probe, with optimal views of the LAA originating with the catheter in the right ventricular inflow tract, right ventricular outflow tract, or pulmonary artery all reported. Occasionally, coronary sinus cannulation is necessary to adequately visualize the LAA in its entirety. Further complicating the decision to use ICE as a screening tool, vascular access is necessary to advance the ICE catheter to the right position, a concept that is not without inherent risk in a patient who may have been otherwise screened using less invasive means. These features collectively suggest ICE imaging should be considered as a complementary approach to LAA thrombus exclusion rather than as a reliable upfront technique.

Although promising in terms of future development, alternative imaging techniques currently remain incompletely validated against the gold standard of TEE, with conflicting data present in the published literature, and their role is currently as second-line therapy (in the case of ICE) or without clear guidance (ce-MDCT) from the joint HRS/EHRA/SOLAECE/APHRS/ECAS expert consensus statement on AF ablation.^4^

### Summary of the current guidelines

In the most recently published joint HRS/EHRA/SOLAECE/APHRS/ECAS expert consensus statement on AF ablation, the role for LAA screening prior to AF ablation remains controversial and is left up to the provider. This is primarily a result of the lack of robust prospective clinical trials assessing which patients can effectively be excluded from LAA screening. A consensus of the current data prompted the writing committee to issue a class 2A recommendation for performing a TEE scan in both patients who present in AF and who have been effectively anticoagulated for three weeks or longer and in those who present in sinus rhythm and who have not been on any anticoagulation prior to the procedure. Patients who present in sinus rhythm on effective oral anticoagulation for three weeks or more received no formal recommendation due to the lack of relevant data. Class 1 recommendations were given to the performance of AF ablation on uninterrupted therapeutic warfarin or dabigatran (1A) and uninterrupted rivaroxaban (1B). The uninterrupted approach was not addressed with regards to screening for LAA thrombus prior to the planned procedure. However, the writing committee acknowledged a rate of 1.6% to 2.1% for thrombus or “sludge” in the LAA in patients presenting for AF ablation who were therapeutically anticoagulated in three separate studies.^5,10,11^

### Clinical scenarios and our approaches based on current literature

Because of the inconsistency in LA/LAA screening and anticoagulation strategies in the studies addressing predictive risk factors, the prevalence of thrombus, and the avoidance of thromboembolic complications, there are several clinical scenarios in existence that clinicians may be required to navigate without clear support from published guidelines or current literature. In patients who present for AF ablation in sinus rhythm on three weeks of OAC therapy with a low risk for LA/LAA thrombus (ie, CHA_2_DS_2_-VASc score = 0 or 1 without renal or structural heart disease), in alignment with the current guidelines, it may be reasonable to forgo LA/LAA screening with imaging before ablation. The role of using alternative risk factors remains understudied in this population, but can help to guide the decision. Patients who present in AF regardless of CHA_2_DS_2_-VASc score should likely undergo LA/LAA thrombus screening, as the majority of published data report a low but persistent incidence of LA/LAA thrombus in this group. Uninterrupted warfarin, dabigatran, and rivaroxaban strategies do not have enough evidence to support forgoing LA/LAA screening based on this parameter alone. Patients who are on anticoagulation and who have undergone prior LA/LAA screening with a negative imaging study within a relatively short time period could reasonably forgo repeat LA/LAA screening if anticoagulation is uninterrupted (ie, there are no missed doses). For those patients undergoing AF ablation on warfarin or NOAC therapy who have had one to two doses withheld before ablation, screening the LA/LAA prior to their procedure may be beneficial until more data exist to address this population and because of the rapid pharmacokinetics and loss of anticoagulant effect associated with these medications.

## Discussion and future directions

The totality of largely retrospective cohort studies with significantly variable strategies in upfront anticoagulation and LA/LAA thrombus screening approaches are inconclusive and in need of prospective studies to standardize treatment. Patients who present in AF and those with elevated risk factors for the development of LA/LAA thrombus remain at elevated risk for the presence of thrombus despite anticoagulation agent or strategy. Patients in sinus rhythm with a CHADS_2_ or CHA_2_DS_2_-VASc score of 0 or 1 without other incrementally validated noninvasive risk factors may be at a very low risk for the presence of LA/LAA thrombus, potentially identifying a population that could forgo screening prior to an AF ablation. However, the presence of any of the aforementioned non-CHA_2_DS_2_-VASc risk factors may warrant caution before deciding to forgo screening, and this is an area for future study. The development of standardized protocols for delayed ce-MDCT to further improve negative predictive values may prove useful for the electrophysiologist, as more procedures are being done on uninterrupted anticoagulation, leaving no subtherapeutic time between the LA/LAA evaluation and the procedure, especially with the promise of reversal agents for the NOACs that are becoming available, resulting in the likely consequence of more ablation procedures being performed on uninterrupted anticoagulation.

As quickly as we have seen a remarkable change in the anticoagulation options available to the clinician, reversible agents soon followed, with the release of agents for all of the available NOACs hopefully on the horizon. A theoretical issue worth discussing exists among electrophysiologists who perform ablation on uninterrupted anticoagulation with no withheld doses of OAC. The aforementioned cohort studies involving uninterrupted dabigatran and rivaroxaban validate the strategy of uninterrupted NOAC therapy against warfarin [with the Apixaban Evaluation of Interrupted or Uninterrupted Anticoagulation for Ablation of AF (AEIOU) study results pending]. These studies show either a decreased bleeding risk with no major increase in complication rates or no significant difference in complications when compared with warfarin. At the current time, however, we lack data on how this fits into AF ablation outcomes in the long-term. One could posit that, if no anticoagulation reversal agent exists, one might be more cautious in identifying endpoints to ablation, especially in those individuals who did not present in AF, wherein the ablation endpoints are less robust than in those who presented in AF. It would be wise, then, to follow these patients forward in registries, specifically looking not only for complications but also for AF recurrences, to ensure that we are not sacrificing procedural efficacy when alternative options exist.

## Figures and Tables

**Table 1:** Relevant Studies Addressing the Incidence of LA/LAA Thrombus Prior to AF Ablation

Study	Design	Preablation OAC Regimen	LA/LAA Thrombus Rate	Main Findings		Other Significantly Associated or Predictive Risk Factors		Author Conclusion(s)
He et al.^3^	Prospective, randomized, involving two groups of patients on no pre-OAC (group 1) and three weeks of OAC (group 2) regimens, respectively; China (n = 188)	Group 1: no OAC pre-AF ablation Group 2: OAC for three weeks with warfarin plus bridging	Group 1: 11.7% Group 2: 6.3%	•	Lower but still significant event rate in group 2	•	Higher bleeding in group 2 (5.3% versus 0% in group 1, p < 0.05)	Anticoagulation does not resolve all thrombi
Puwanant et al.^5^	Retrospective review; USA (n = 1,058)	Warfarin or uninterrupted OAC	63/1058 (0.6%)	•	Rate of LAA/LAA thrombus increased with increasing CHADS_2_ score	•	For those with CHADS_2_ score = 0, 0% had LA/LAA thrombus, chronic heart failure and low left ventricular ejection fraction	If CHADS_2_ score = 0, type of AF is persistent, and no OAC was given previously, then TEE should be performed
Anselmino et al.^7^	Retrospective, multi-left; Italy (n = 1,539)	Physician-dependent	12/1,539	•	Ten of the 12 patients were on warfarin	•	First AF procedure	TEE could potentially be avoided in patients presenting for first AF ablation in sinus rhythm with CHA_2_DS_2_-VASc score = 0 or 1
				•	All of the LAA thrombus patients with CHA_2_DS_2_-VASc score = 0 or 1 presented in AF			
				•	All of the LAA thrombus patients who presented in sinus rhythm had CHA_2_DS_2_-VASc score ≥ 2			
Calvo et al.^8^	Prospective; Spain (n = 408)	Warfarin with LMWH bridge	6/408 (1.47%)	•	Five of the six patients had CHADS_2_ score < 2	•	Persistent AF	TEE may not be necessary in patients with persistent AF and no LA dilation or structural cardiomyopathy
				•	All patients with LAA thrombus had persistent AF with LA dilation	•	Female gender	
						•	Structural heart disease	
						•	LA dilation	
Yamashita et al.^9^	Retrospective, single-left; Japan (n = 446)	According to Japanese Society Guidelines, continued until the day of the procedure	13/446 (2.9%)	•	Advanced age persistent AF, and structural heart disease were predictors of LAA thrombus	•	0 of 136 lone AF patients under the age of 60 years had LAA thrombus	It may be reasonable to omit TEE in young, paroxysmal lone AF patients
McCready et al.^10^	Prospective, single-left; UK (n = 635)	Warfarin with LMWH bridge	12/635 (1.9%)	•	Larger LA, persistent AF, hypertension, age > 75 years, cardiomyopathy	•	No thrombus was found in the patients with no risk factors	The performance of TEE may be unnecessary in patients who present without risk factors
Scherr et al.^11^	Prospective, single-left registry; USA (n = 732)	Warfarin with LMWH bridge	12/732 (1.6%)	•	CHADS_2_ score ≥ 2, larger LA diameter	•	Those with CHADS_2_ score = 0 had 0.3% incidence	The LAA thrombus rate is low in patients with CHADS_2_ score = 0 and LA diameter < 4.5 cm
Wallace et al.^12^	Retrospective, single left; USA (n = 192)	According to practice guidelines, bridged with LMWH	7/192 (3.6%)	•	Structural heart disease, LA dilation, and number of AF ablations	•	Three of seven patients had normal LV function and paroxysmal AF	It is suggested that all patients undergo TEE prior to AF ablation
						•	Two with paroxysmal AF presented in sinus rhythm; both had CHADS_2_ = 2	
Wyrembak et al.^28^	Retrospective, single-left; USA (n = 937)	Patients were separated into two groups of OAC: warfarin (group 1; n = 517) and NOAC (group 2, n = 420)	Group 1: 8/517 (1.55%), 7/8 with INR > 2 Group 2: 1/420 (0.24%)	•	Those taking NOACs had a lower incidence of LAA thrombus	•	Heart failure	Treatment with NOAC is associated with a lower incidence of LAA thrombus pre-AF ablation
						•	Diabetes	
						•	Higher CHA_2_DS_2_-VASc	
						•	Lower ejection fraction	
						•	Lower LAA velocity	
						•	Presence of spontaneous echo contrast	
Nishikii et al.^34^	Retrospective; Japan (n = 543)	All patients on warfarin (INR: 2–3 if aged < 70 years, INR: 1.6–2.6 if aged > 70 years)	6.4% LAA thrombus	•	Higher incidence of LAA thrombus associated with higher CHADS_2_ score	•	LA volume > 50 ml	The use of additional radiofrequency to perform risk stratification in patients with low CHADS_2_ scores is suggested
				•	2.1% of patients with CHADS_2_ score = 0 or 1 had LAA thrombus	•	Ejection fraction < 56%	
						•	Brain natriuretic peptide > 75 pg/ml	
Zoppo et al.^35^	All patients were effectively anticoagulated prior; Italy (n = 430)	Warfarin with LMWH bridge	10/430 (2/2%)	•	Higher CHADS_2_ or CHA_2_DS_2_-VASc score	•	No patients with CHADS_2_ = 0 had LAA thrombus	Despite OAC use, a 2.3% LAA thrombus rate occurred
				•	Larger LA size			
Michael et al.^36^	National survey involving two groups (routine TEE or selective TEE); Canada (n = 2,225)	Variable	11/2225 (0.49%)	•	Selective TEE did not improve ability to detect LAA thrombus	•	None	Low-risk patients in a cohort may not benefit from pre-procedural TEE

OAC: oral anticoagulation; LA: left atrial/atrium; LAA: left atrial appendage; TEE: transesophageal echocardiography; AF: atrial fibrillation; n: number; LMWH: low-molecular-weight heparin; NOAC: novel oral anticoagulant; INR: international normalized ratio.

## References

[1] Knight BP (2009). Transesophageal echocardiography before atrial fibrillation ablation: looking before cooking. J Am Coll Cardiol..

[2] Wazni OM, Rossillo A, Marrouche NF (2005). Embolic events and char formation during pulmonary vein isolation in patients with atrial fibrillation: impact of different anticoagulation regimens and importance of intracardiac echo imaging. J Cardiovasc Electrophysiol..

[3] He H, Kang J, Tao H (2009). Conventional oral anticoagulation may not replace prior transesophageal echocardiography for the patients with planned catheter ablation for atrial fibrillation. J Interv Card Electrophysiol..

[4] Calkins H, Hindricks G, Cappato R (2017). 2017 HRS/EHRA/ECAS/APHRS/SOLAECE expert consensus statement on catheter and surgical ablation of atrial fibrillation. Heart Rhythm..

[5] Puwanant S, Varr BC, Shrestha K (2009). Role of the CHADS2 score in the evaluation of thromboembolic risk in patients with atrial fibrillation undergoing transesophageal echocardiography before pulmonary vein isolation. J Am Coll Cardiol..

[6] Sikorska A, Baran J, Pilichowska-Paszkiet E (2015). Risk of left atrial appendage thrombus in patients scheduled for ablation for atrial fibrillation: beyond the CHA2DS2VASc score. Pol Arch Med Wewn..

[7] Anselmino M, Garberoglio L, Gili S (2017). Left atrial appendage thrombi relate to easily accessible clinical parameters in patients undergoing atrial fibrillation transcatheter ablation: a multicenter study. Int J Cardiol..

[8] Calvo N, Mont L, Vidal B (2011). Usefulness of transoesophageal echocardiography before circumferential pulmonary vein ablation in patients with atrial fibrillation: is it really mandatory?. Europace..

[9] Yamashita E, Takamatsu J, Tada J (2010). Transesophageal echocardiography for thrombus screening prior to left atrial catheter ablation. Circ J..

[10] McCready JW, Nunn L, Lambiase PD (2010). Incidence of left atrial thrombus prior to atrial fibrillation ablation: is pre-procedural transoesophageal echocardiography mandatory?. Europace..

[11] Scherr D, Dalal D, Chilukuri K (2009). Incidence and predictors of left atrial thrombus prior to catheter ablation of atrial fibrillation. J Cardiovasc Electrophysiol..

[12] Wallace TW, Atwater BD, Daubert JP (2010). Prevalence and clinical characteristics associated with left atrial appendage thrombus in fully anticoagulated patients undergoing catheter-directed atrial fibrillation ablation. J Cardiovasc Electrophysiol..

[13] Doukky R, Garcia-Sayan E, Patel M (2016). Impact of diastolic function parameters on the risk for left atrial appendage thrombus in patients with nonvalvular atrial fibrillation: a prospective study. J Am Soc Echocardiogr..

[14] Zeng WT, Sun XT, Tang K (2015). Risk of thromboembolic events in atrial fibrillation with chronic kidney disease. Stroke..

[15] Wazni OM, Beheiry S, Fahmy T (2007). Atrial fibrillation ablation in patients with therapeutic international normalized ratio: comparison of strategies of anticoagulation management in the periprocedural period. Circulation..

[16] Bassiouny M, Saliba W, Rickard J (2013). Use of dabigatran for periprocedural anticoagulation in patients undergoing catheter ablation for atrial fibrillation. Circ Arrhythm Electrophysiol..

[17] Bin Abdulhak AA, Khan AR, Tleyjeh IM (2013). Safety and efficacy of interrupted dabigatran for peri-procedural anticoagulation in catheter ablation of atrial fibrillation: a systematic review and meta-analysis. Europace..

[18] Hohnloser SH, Camm AJ (2013). Safety and efficacy of dabigatran etexilate during catheter ablation of atrial fibrillation: a meta-analysis of the literature. Europace..

[19] Phan K, Wang N, Pison L, Kumar N, Hitos K, Thomas SP (2015). Meta-analysis of dabigatran vs warfarin in patients undergoing catheter ablation for atrial fibrillation. In J Cardiol..

[20] Winkle RA, Mead RH, Engel G, Kong MH, Patrawala RA (2014). Peri-procedural interrupted oral anticoagulation for atrial fibrillation ablation: comparison of aspirin, warfarin, dabigatran, and rivaroxaban. Europace..

[21] Lakkireddy D, Reddy YM, Di Biase L (2012). Feasibility and safety of dabigatran versus warfarin for periprocedural anticoagulation in patients undergoing radiofrequency ablation for atrial fibrillation: results from a multicenter prospective registry. J Am Coll Cardiol..

[22] Providência R, Marijon E, Albenque JP (2014). Rivaroxaban and dabigatran in patients undergoing catheter ablation of atrial fibrillation. Europace..

[23] Winkle RA, Mead RH, Engel G, Kong MH, Patrawala RA (2014). Peri-procedural interrupted oral anticoagulation for atrial fibrillation ablation: comparison of aspirin, warfarin, dabigatran, and rivaroxaban. Europace..

[24] Armbruster HL, Lindsley JP, Moranville MP (2015). Safety of novel oral anticoagulants compared with uninterrupted warfarin for catheter ablation of atrial fibrillation. Ann Pharmacother..

[25] Cappato R, Marchilinski FE, Hohnloser SH (2015). Uninterrupted rivaroxaban vs. uninterrupted vitamin K antagonists for catheter ablation in non-valvular atrial fibrillation. Eur Heart J..

[26] Calkins H, Willems S, Gerstenfeld DP (2017). Uninterrupted dabigatran versus warfarin for ablation in atrial fibrillation. N Engl J Med..

[27] Gula LJ, Massel D, Redfearn DP (2010). Impact of routine transoesophageal echocardiography on safety, outcomes, and cost of pulmonary vein ablation: inferences drawn from a decision analysis model. Europace..

[28] Wyrembak J, Campbell K, Steinberg B (2017). Incidence and predictors of left atrial appendage thrombus in patients treated with nonvitamin K oral anticoagulants versus warfarin before catheter ablation for atrial fibrillation. Am J Cardiol..

[29] Billchick KC, Mealor A, Gonzalez J (2016). Effectiveness of integrating delayed computed tomography angiography imaging for left atrial appendage thrombus exclusion into the care of patients undergoing ablation of atrial fibrillation. Heart Rhythm..

[30] Romero J, Husain SA, Kelesidis I (2013). Detection of left atrial appendage thrombus by cardiac computed tomography in patients with atrial fibrillation: a meta-analysis. Circ Cardiovasc Imaging..

[31] Lazoura O, Ismail TF, Pavitt C (2016). A low-dose, dual-phase cardiovascular CT protocol to assess left atrial appendage anatomy and exclude thrombus prior to left atrial intervention. Int J Cardiovasc Imaging..

[32] Sriram CS, Banchs JE, Moukabary T, Moradkhan R, Gonzalez MD (2015). Detection of left atrial thrombus by intracardiac echocardiography in patients undergoing ablation of atrial fibrillation. J Interv Card Electrophysiol..

[33] Ren JF, Marchlisnki FE, Supple GE (2013). Intracardiac echocardiographic diagnosis of thrombus formation in the left atrial appendage: a complementary role to transesophageal echocardiography. Echocardiography..

[34] Nishikii-Tachibana M, Murakoshi N, Seo Y (2015). Prevalence and clinical determinants of left atrial appendage thrombus in patients with atrial fibrillation before pulmonary vein isolation. Am J Cardiol..

[35] Zoppo F, Brandolino G, Berton A (2012). Predictors of left atrium appendage clot detection despite on-target warfarin prevention for atrial fibrillation. J Interv Card Electrophysiol..

[36] Michael KA, Redfearn DP, Baranchuk A (2009). Transesophageal echocardiography for the prevention of embolic complications after catheter ablation for atrial fibrillation. J Cardiovasc Electrophysiol..

